# Gold nanoparticles supported on mesoporous silica: origin of high activity and role of Au NPs in selective oxidation of cyclohexane

**DOI:** 10.1038/srep18817

**Published:** 2016-01-05

**Authors:** Pingping Wu, Peng Bai, Zifeng Yan, George X. S. Zhao

**Affiliations:** 1 State Key Laboratory of Heavy Oil Processing, PetroChina Key Laboratory of Catalysis, School of Chemical Engineering, China University of Petroleum, Qingdao, China, 266580; 2 School of Chemical Engineering, The University of Queensland, St Lucia, 4072.

## Abstract

Homogeneous immobilization of gold nanoparticles (Au NPs) on mesoporous silica has been achieved by using a one-pot synthesis method in the presence of organosilane mercapto-propyl-trimethoxysilane (MPTMS). The resultant Au NPs exhibited an excellent catalytic activity in the solvent-free selective oxidation of cyclohexane using molecular oxygen. By establishing the structure-performance relationship, the origin of the high activity of mesoporous supported Au catalyst was identified to be due to the presence of low-coordinated Au (0) sites with high dispersion. Au NPs were confirmed to play a critical role in the catalytic oxidation of cyclohexane by promoting the activation of O_2_ molecules and accelerating the formation of surface-active oxygen species.

Cyclohexanol and cyclohexanone (also known as K/A oil) can be produced by selective oxidation of cyclohexane, which are important chemical intermediates for the bulk production of polyamide and plastics, such as Nylon 6 and Nylon 6, 6. Transition metal salts such as Mn and Co naphthenates are the catalysts commonly used in the industry. In practice, the cyclohexane conversion is limited to be less than 5% to prevent the formation of excessive amounts of by-products, giving 70–85% selectivity towards K/A oil. However, these homogeneous catalysts are difficult to be separated from the reaction mixture, arousing engineering and environmental concerns.

Gold (Au) nanoparticles (NPs) are catalytically active in many important oxidation reactions^1^,^2^,^3^,^4^,^5^,^6^,^7^. Supported Au NPs catalysts, such as Au/ZSM-5^8^, Au/MCM-41^9^ and Au@TiO_2_/MCM-22^10^ have been used in cyclohexane oxidation with a K/A oil selectivity as high as 90%. Many research groups have separately reported their findings on supported Au NPs catalysts with similar results e.g. Au/Al_2_O_3_, Au/C^11^,^12^. In our previous work^13^, a thiol-containing organosilane, mercapto-propyl-trimethoxysilane (MPTMS), was used in the synthesis of mesoporous silica supported fine Au NPs (2–4 nm) through a simple one-pot method. The resultant Au NPs exhibited an excellent catalytic activity and high stability in the solvent-free selective oxidation of cyclohexane using molecular oxygen. However, the origin of the high activity of the catalyst and the role of Au NPs in the activation of cyclohexane were not well addressed.

With regard to the role of Au NPs in the aerobic oxidation of cyclohexane, Hereijgers *et al.* held a completely different opinion, who reported that Au was not active in cyclohexane oxidation at all and the reaction proceeded via a radical-chain mechanism, characteristic of autoxidation^14^. However, in a subsequent work reported by Liu *et al.*, gold clusters, Au_n_, were found to have high activity (TOF is up to 18500 h^−1^) and high K/A oil selectivity (>90%) in the aerobic oxidation of cyclohexane^15^. No conversion was observed in the absence of Au_n_ clusters. The activity of Au_n_ clusters was observed to have a volcano-type dependence on their size with the maximum at n = 39. Hence, the role of Au NPs in the oxidation of cyclohexane remains unclear and is still open to debate^16^.

In this work, the origin of the high activity of Au NP_S_ supported on mesoporous silica was explored by correlating the catalytic performance with the structural properties of Au catalysts prepared under different conditions. The role of Au NPs in the oxidation of cyclohexane was investigated by conducting a simple control experiment and the activation of O_2_ molecules by Au NPs was examined by using a O_2_-temperature programmed desorption (O_2_-TPD) technique. Based on the understanding of the catalytic mechanism of Au NPs in the oxidation of cyclohexane, the effect of reaction conditions was further investigated and optimized.

## Results and Discussion

**Figure 1:**
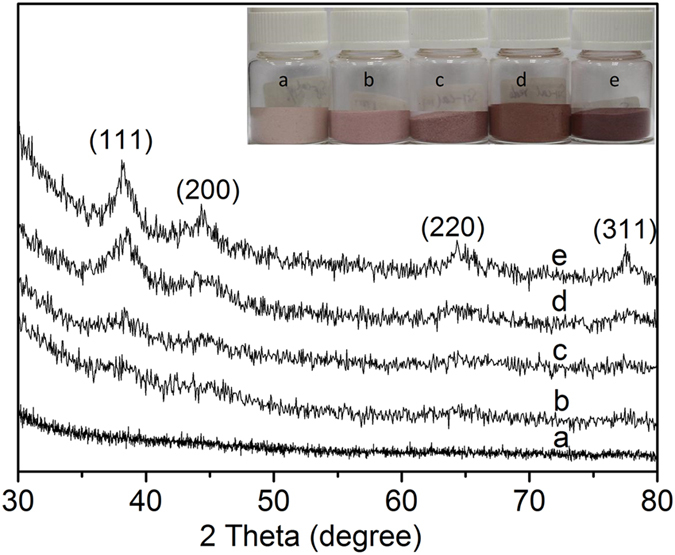
XRD patterns of catalysts (**a**) 0.2 *wt.*%Au/MPTMS-SiO_2_-cal, (**b**) 0.4 *wt.*%Au/MPTMS-SiO_2_-cal, (**c**) 0.95 *wt.*%Au/MPTMS-SiO_2_-cal and (**d**) 1.2 *wt.*%Au/MPTMS-SiO_2_-cal and (**e**) 1.6 *wt.*% Au/MPTMS-SiO_2_-cal.

Figure 1 shows the XRD patterns of Au catalysts with different gold loadings. When the Au loading was less than 1 *wt.*%, very weak or even no diffraction peaks were detected, which is due to very small Au particles and/or a low concentration of Au on the catalyst. While on catalysts with Au loadings of more than 1 *wt.*%, four peaks at 38.18, 44.43, 64.55 and 77.75^o^ two theta were observed, which are assigned to cubic Au nanoparticles (JCPDS card no.: 4–784). With the increase of Au loading, the color of resultant catalysts changed from light pink to ruby (inset in Fig. 1).

The N_2_ adsorption/desorption isotherms of different Au loading catalysts are shown in Fig. S1, the textural properties and Au metal dispersions on different Au loading catalysts were summarized in Table 1. It is seen from Fig. S1 that all catalysts displayed a type IV isotherm with a hysteresis loop. The pore size distribution centered in the range of 3.5–6 nm; however, the pore size distribution became wider with the increase of Au loading. This is due to the distortion effect caused by the immobilization of Au NPs in the pore surface. No obvious difference was observed on the textural properties of these Au catalysts because a little amount of Au precursor introduced has no obvious effect on the mesoporous structure of silica support. However, the metal dispersion decreased with the increase of Au loading (shown in Table 1).

**Figure 2:**
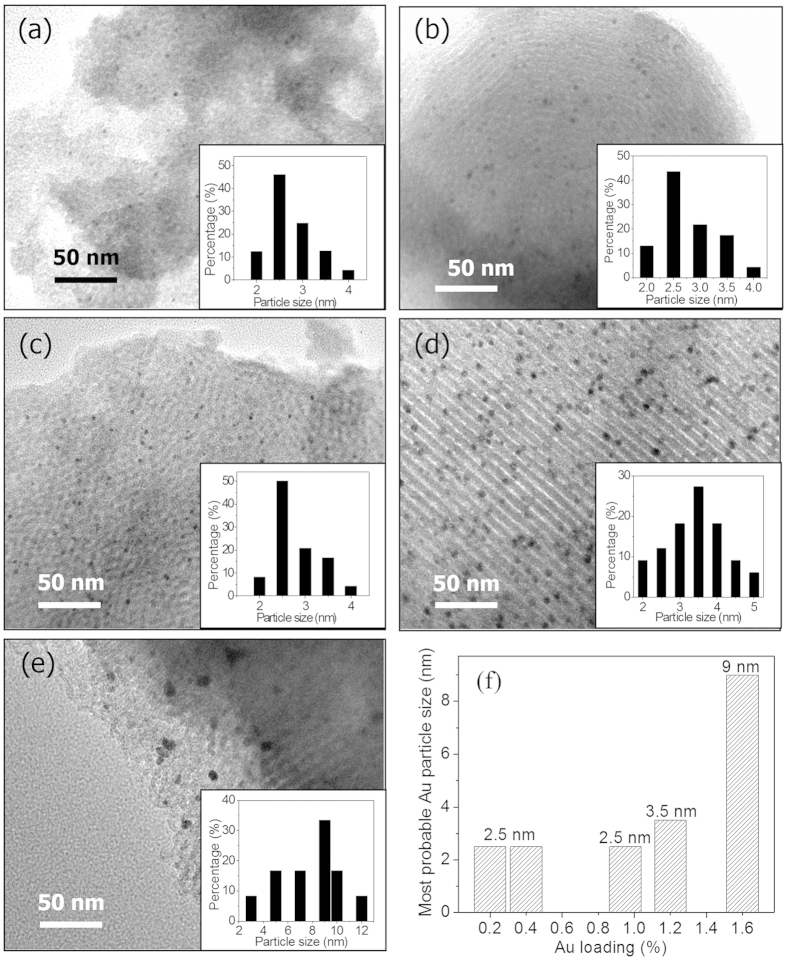
TEM images of catalysts (**a**) 0.2 *wt.*%Au/MPTMS-SiO_2_-cal, (**b**) 0.4 *wt.*%Au/MPTMS-SiO_2_-cal, (**c**) 0.95 *wt.*%Au/MPTMS-SiO_2_-cal and (**d**) 1.2 *wt.*%Au/MPTMS-SiO_2_-cal and (**e**) 1.6 *wt.*% Au/MPTMS-SiO_2_-cal, (**f**) the most probable distribution of Au particle size on different Au loading catalysts.

**Figure 3:**
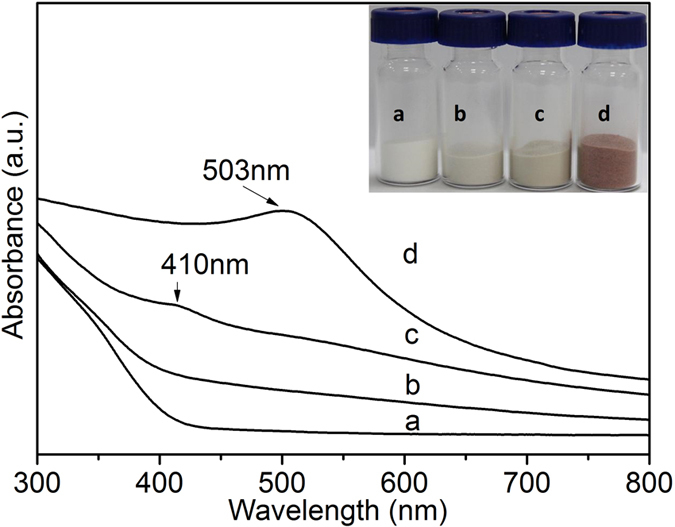
UV-vis spectra of catalysts before and after reduction (**a**) Au/MPTMS-SiO_2_-as, (**b**) Au/MPTMS-SiO_2_-BH_4_, (**c**) Au/MPTMS-SiO_2_-H_2_ and (**d**) Au/MPTMS-SiO_2_-cal.

Figure 2 shows the TEM images of Au catalysts with different Au loadings. It was found that when the Au loading was less than 1.2 *wt.*%, the size distributions of Au NPs were in the similar range of 2–5 nm. With the Au loading increasing up to 1.6 *wt.*%, the Au NPs began to aggregate to larger ones (3–12 nm). In this work all catalysts were prepared with a MPTMS/TEOS ratio of *ca.* 1/9, under which the amount of Au loading was optimized and the results showed that with an Au loading of less than 1.0 *wt.*%, the well dispersed Au NPs in the range of 2–5 nm were obtained. From Fig. 3f, it was observed that the most probable distribution of Au NPs was 2.5 nm on catalysts 0.2 *wt.*%Au/MPTMS-SiO_2_-cal, 0.4 *wt.*%Au/MPTMS-SiO_2_-cal and 0.95 *wt.*%Au/MPTMS-SiO_2_-cal, and increased to 3.5 nm and 9 nm on catalysts 1.2 *wt.*%Au/MPTMS-SiO_2_-cal and 1.6 *wt.*%Au/MPTMS-SiO_2_-cal, respectively.

Different reduction methods were applied to prepare Au NPs catalyst with different Au chemical status. Catalyst Au/MPTMS-SiO_2_-cal was prepared from as-synthesized sample Au/MPTMS-SiO_2_-as while catalysts Au/MPTMS-SiO_2_-BH_4_ and Au/MPTMS-SiO_2_-H_2_ were prepared from sample Au/MPTMS-SiO_2_-3et after template removal by ethanol extraction. The structural properties of different catalysts are listed in Table 2. Catalyst Au/MPTMS-SiO_2_-cal exhibited the largest surface area (862 m^2^/g) and pore volume (0.73 m^3^/g), due to the complete removal of surfactant and functional groups. Sample Au/MPTMS-SiO_2_-3et after template removal by ethanol extraction showed relative lower specific surface area (642 m^2^/g) and pore volume (0.78 m^3^/g) than that of catalyst Au/MPTMS-SiO_2_-cal which is due to the incomplete removal of surfactant^17^. However, after NaBH_4_ or H_2_ reduction, the surface area and pore volume decreased dramatically, especially for catalyst Au/MPTMS-SiO_2_-BH_4_ with a specific surface area of 328 m^2^/g and the pore volume of 0.31 cm^3^/g. This significant decrease may be attributed to the formation of Au clusters after reduction and release of bonded mercaptopropyl groups (-CH_2_-CH_2_-CH_2_-SH), both Au clusters and released mercaptopropyl groups may block the pores of mesoporous silica support. Minor decrease occurred on catalyst Au/MPTMS-SiO_2_-H_2_ with a specific surface area of 438 m^2^/g and the pore volume of 0.48 m^3^/g, which may be due to the partial decomposition of functional groups on catalyst Au/MPTMS-SiO_2_-H_2_ during the H_2_ reduction at 250 °C.

The Au contents in different catalysts were measured by using the inductive-coupled plasma-mass spectrometer (ICP-MS) technique and the results are shown in Table 2. The Au loading efficiencies on catalysts Au/MPTMS-SiO_2_-BH_4_, Au/MPTMS-SiO_2_-H_2_ and Au/MPTMS-SiO_2_-cal were 67%, 81% and 95%, respectively. It can be concluded that the catalyst reduced by high temperature calcination provided the highest Au loading efficiency, while the low Au loading efficiency on catalysts Au/MPTMS-SiO_2_-BH_4_ and Au/MPTMS-SiO_2_-H_2_ may be due to the leaching of Au species during the template removal process and/or reduction process.

The interaction of gold species with thiol groups on catalysts reduced by different methods were investigated to reveal the active gold species during cyclohexane oxidation. The evidence for the incorporation of organosiloxanes with thiol group into the silica framework of catalysts Au/MPTMS-SiO_2_-BH_4_ and Au/MPTMS-SiO_2_-H_2_ was obtained from ^29^Si and ^13^C CP MAS NMR results shown in Fig. S2. The interaction of gold species with thiol groups on different catalysts were characterized by UV-vis spectroscopy and X-ray photoelectron spectroscopy (XPS), with respect to the Au cluster size and chemical state.

As can be seen in Fig. 3, catalyst Au/MPTMS-SiO_2_-BH_4_ showed similar absorption spectrum with the as-synthesized sample Au/MPTMS-SiO_2_-as. The UV-vis spectrum of catalyst Au/MPTMS-SiO_2_-H_2_ exhibited a weak absorption peak at ca. 410 nm, which reminds us of the spectra of thiolate-passivated Au clusters (<2 nm), which showed peaks in the range of 300 ~ 450 nm^18^,^19^. Thus, the enhanced UV-vis adsorption in the visible range of catalysts Au/MPTMS-SiO_2_-BH_4_ may be also due to the formation of thiolate stabilized Au clusters after reduction. The further enhancement of adsorption in the lower energy range on catalyst Au/MPTMS-SiO_2_-H_2_ is probably due to the formation of bigger-sized Au clusters than that on catalyst Au/MPTMS-SiO_2_-BH_4_ 20, and/or weak interaction between Au clusters and thiol functional groups because of partial rupture of S-Au bonds under the H_2_ reduction. These features are completely different from that of the nanometer-sized gold particles, the UV-vis spectrum of which has an obvious absorption band at ca. 500 nm (surface Plasmon resonance band of Au NPs). The plasmon of nanoparticle gold is normally at about 520 nm. The blue shift on catalyst Au/MPTMS-SiO_2_-cal is probably due to smaller Au NPs size (<2 nm) and/or interaction between Au NPs and silica matrix. While the blue shift on catalysts Au/MPTMS-SiO_2_-BH_4_ and Au/MPTMS-SiO_2_-H_2_ (from 500 nm to 410 nm) is due to the strong interaction between thiol groups and Au clusters in thiolate-passivated Au clusters, these deduction was further confirmed by XPS data. The different color observed on different catalysts (see Fig. 3, inset) provided a direct evidence of the distinct chemical status of Au species on different catalysts.

**Figure 4:**
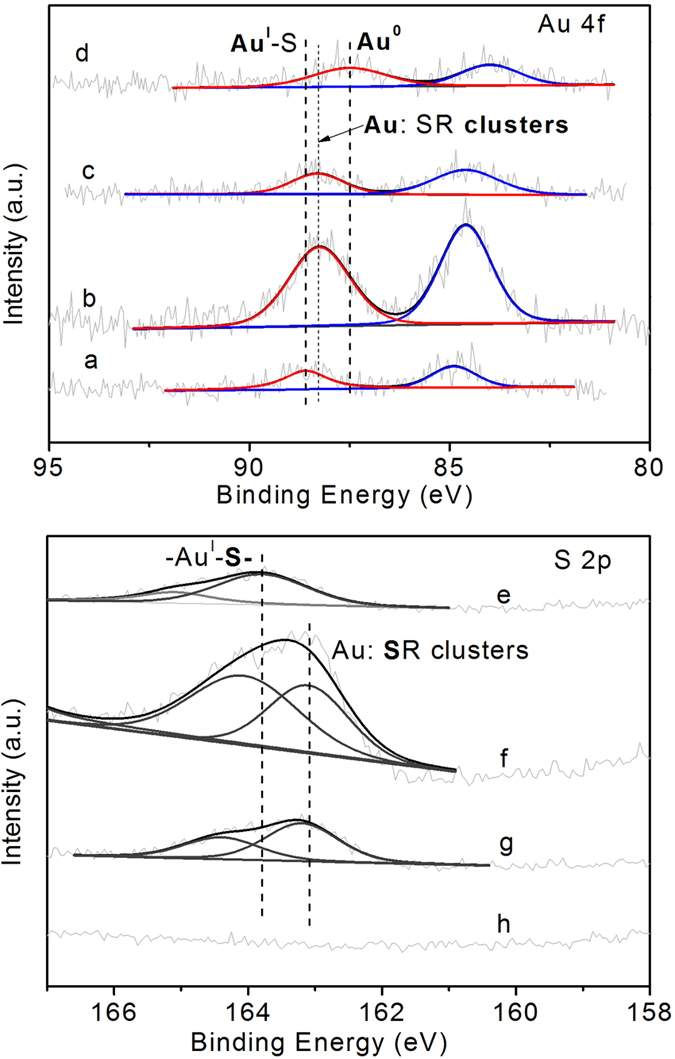
Au (4f) and S (2p) XPS spectra of catalysts (**a**,**e**) Au/MPTMS-SiO_2_-as, (**b**,**f**) Au/MPTMS-SiO_2_-BH_4_, (**c**,**g**) Au/MPTMS-SiO_2_-H_2_ and (**d**,**h**) Au/MPTMS-SiO_2_-cal.

**Figure 5:**
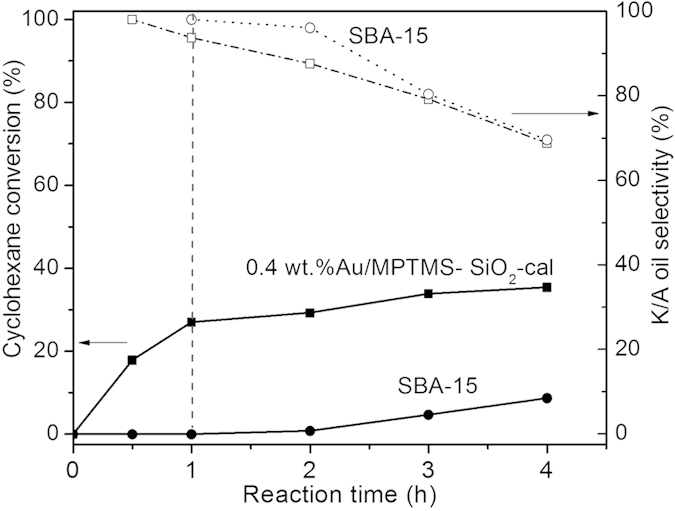
Control experiment results on SBA-15 and catalyst 0.4 *wt.*%Au/MPTMS- SiO_2_-cal.

XPS technique was utilized to study the chemical combination status of Au and S species in the as-synthesized and reduced catalysts. In Fig. 4a, Au 4 f_7/2_ and 4 f_5/2_ peaks with binding energy of 84.9 eV and 88.5 eV in sample Au/MPTMS-SiO_2_-as were observed which is corresponding to Au (I) species in a gold thiolate complex (84.9 eV and 88.5 eV)^21^. This indicated that the Au species presented as [-Au^I^SR-]_n_ polymer in sample Au/MPTMS-SiO_2_-as. After H_2_ or NaBH_4_ reduction, the Au 4 f binding energy was determined to be ca. 84.5 eV and 88.3 eV (Fig. 4b,c), which is slightly higher than that of bulk gold (such as 84.0 and 87.7 eV) but at the similar position of thiolate-passivated Au clusters (Au: SR clusters) at 84.4 eV^22^. This implies that gold thiolate complexes [-Au^I^SR-]_n_ were reduced to form thiolate-passivated Au clusters after NaBH_4_ or H_2_ reduction. After high temperature calcination, Au 4 f_7/2_ and 4 f_5/2_ with binding energy of 84.0 eV and 87.6 eV were observed on catalyst Au/MPTMS-SiO_2_-cal (Fig. 5d), which are typical values for bulk Au^0^ (84.0 eV and 87.7 eV), indicating the presence of metallic Au after high temperature calcination.

The S 2p core level photoelectron spectra of catalysts reduced by different methods are displayed in Fig. 4(e–h). The S 2p doublet peaks with binding energy (BE) of 163.8 eV and 165.1 eV were observed in sample Au/MPTMS-SiO_2_-as. The main peak at 163.8 eV was found at the same position of the S 2p_3/2_ signal of [-Au^I^SR-]_n_ thiolate complexes, confirming the formation of chemical bond between S and Au (I) during synthesis^18^. Upon reduction, the S 2p signals were significantly shifted to lower bonding energy of *ca.* 163.1 eV on catalyst Au/MPTMS-SiO_2_-BH_4_ and *ca.* 163.2 eV on catalyst Au/MPTMS-SiO_2_-H_2_. For reference, the binding energy of S (2p_3/2_) on thiolate-passivated Au clusters ranges from 162.0 to 162.3 eV^23^. Thus, this shift towards lower BE is due to the formation of thiolate-passivated Au clusters. The BE of S (2p_3/2_) on the catalysts prepared in this work is larger than the reference data, suggesting the existence of some amount of gold thiolate complexes [-Au^I^SR-]_n_ during the reduction^24^. No S 2p peaks were observed on the calcined catalyst because the complete removal of functional groups during high temperature calcination. Thus, the XPS results confirmed the presence of thiolate-passivated Au clusters on catalysts Au/MPTMS-SiO_2_-BH_4_ and Au/MPTMS-SiO_2_-H_2_ but metallic Au on catalyst Au/MPTMS-SiO_2_-cal.

The catalytic activity of the synthesized catalyst was evaluated on selective oxidation of cyclohexane with molecular oxygen. The cyclohexane conversion, useful products selectivity and turnover frequency (TOF) are summarized in Table 3. The measured reaction rates are reported as turnover frequencies (TOF) and measured in units of mole of product K/A oil produced per mole of surface gold per hour. As can be seen in Table 3, catalysts 0.4 *wt.*%Au/MPTMS-SiO_2_-cal, 0.95 *wt.*%Au/MPTMS-SiO_2_-cal and 1.2 *wt.*%Au/MPTMS-SiO_2_-cal exhibited similar cyclohexane conversion of 29.7%, 27.4% and 28.3%, respectively, with a similar K/A oil selectivity (>90%). Among these three catalysts with similar cyclohexane conversion, catalyst 0.4 *wt.*%Au/MPTMS-SiO_2_-cal exhibited the highest reaction rate with a TOF of 59951 h^−1^ in a 2 h reaction. Although catalyst 0.2 *wt.*%Au/MPTMS-SiO_2_-cal exhibited a lower conversion of 16.7%, it possessed a remarkably high TOF of 69091 h^−1^, to the best of our knowledge, which is so far the highest TOF reported in literature^15^,^25^. Catalyst 1.6 *wt.*%Au/MPTMS-SiO_2_-cal, despite having the highest Au loading of 1.6 *wt.*% among calcined catalysts, exhibited the lowest cyclohexane conversion (16.4%). Note that the reaction results are closely related to the Au NPs size, the size distribution histograms are shown in Fig. 2. It was observed that the size distribution of Au NPs shifted to larger ones with the increase of Au loading from 0.4 *wt.*% to 1.6 *wt.*%, indicating the agglomeration of Au NPs with the increase of Au loading. This agglomeration results in the decrease of the number of surface Au atoms with low-coordination. This exactly explains that catalysts 0.95 *wt.*%Au/MPTMS-SiO_2_-cal, 1.2 *wt.*% Au/MPTMS-SiO_2_-cal and 1.6 *wt.*%Au/MPTMS-SiO_2_-cal with higher Au loading exhibit lower TOF than that of catalyst 0.4 *wt.*%Au/MPTMS-SiO_2_-cal. Therefore, on catalysts *x*Au/MPTMS-SiO_2_-cal, the most significant factor for the catalytic activity of cyclohexane oxidation is not the amount of Au loaded, but the size of Au NPs^15^, corresponding to the amount of low-coordinated Au atoms.

The reduction method has a great influence on the catalytic activity of Au catalysts for cyclohexane oxidation. It is worth to note that the catalytic activity (TOF) is remarkably high on catalyst Au/MPTMS-SiO_2_-cal (23368 h^−1^) comparing with the other two catalysts. The lower TOF on catalysts Au/MPTMS-SiO_2_-BH_4_ (2592 h^−1^) and Au/MPTMS-SiO_2_-H_2_ (12181 h^−1^) indicated a low catalytic activity which is probably due to the Au (+1) species strongly coordinated with –SH which are very stable^26^. While on catalyst Au/MPTMS-SiO_2_-cal, [-Au^I^SR-]_n_ complexes were completely decomposed to form Au (0) NPs after high temperature calcination. Thus, it can be concluded that low-coordinated Au (0) sites with high dispersion are responsible for the high activity of Au catalysts in the cyclohexane oxidation and gold species coordinated with thiol groups possessed low catalytic activity. This result was confirmed by another work using bis(triethoxysily) propane tetrasulfide (TESPTS) with thioether functionality to stabilize Au NPs^27^.

In order to access whether Au NPs played a role in cyclohexane oxidation, a simple control experiment was carried out by comparing the catalytic performance of the pure SBA-15 material and the SBA-15 supported Au NPs catalyst 0.4 *wt.*%Au/MPTMS-SiO_2_-cal (shown in Fig. 5) under strictly identical conditions. It was found that the pure SBA-15 exhibited no catalytic activity in the first 2 h of reaction, and cyclohexane conversions of about 5% and 8% were observed after 3 h and 4 hour of reaction, respectively. By contrast, a significant higher catalytic activity was observed on catalyst 0.4 *wt.*%Au/MPTMS-SiO_2_-cal with a cyclohexane conversion of 27% after 1^st^ hour of reaction. In a direct observation, the system pressure started to decrease after 20 min and a pressure decrease of about 20 psi was observed after 1 h of reaction when using gold catalyst. However, for the pure SBA-15 sample, no obvious pressure decrease was observed in the first hour of reaction and about 3 psi decrease after 2 h of reaction which was similar with the results in the absence of catalyst. These results confirmed that Au NPs really played an important role in the catalytic oxidation of cyclohexane. It was reported that the cyclohexane oxidation in the absence of catalyst followed the autoxidation mechanism^28^. The similar trend of cyclohexane conversion in the following hours was observed on pure SBA-15 and catalyst 0.4 *wt.*%Au/MPTMS-SiO_2_-cal, indicating that the reaction may follow the autoxidation mechanism. Hence, it means that Au NPs played the critical role in the initiation stage of cyclohexane oxidation, possibly promoting the activation of reactants and/or formation of intermediate cyclohexyl hydroperoxide (CyOOH).

**Figure 6:**
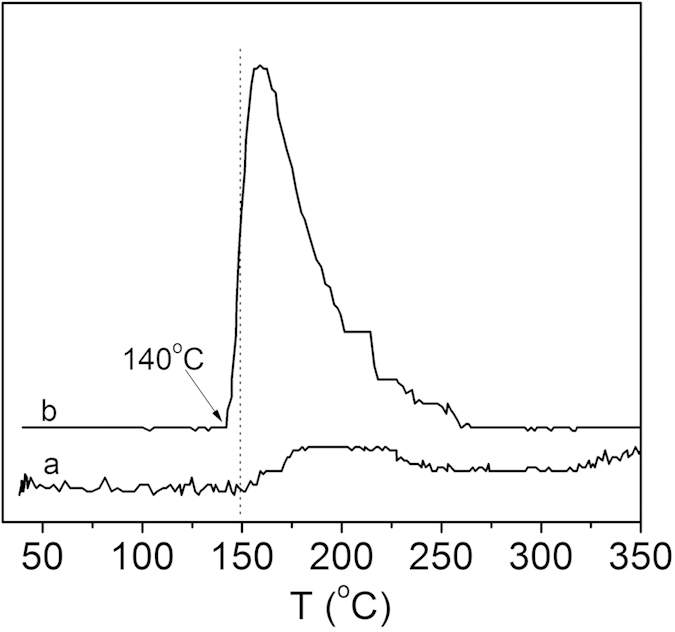
O_2_-TPD profiles of (a) SBA-15 and (b) 0.4 *wt.*%Au/MPTMS- SiO_2_-cal.

**Figure 7:**
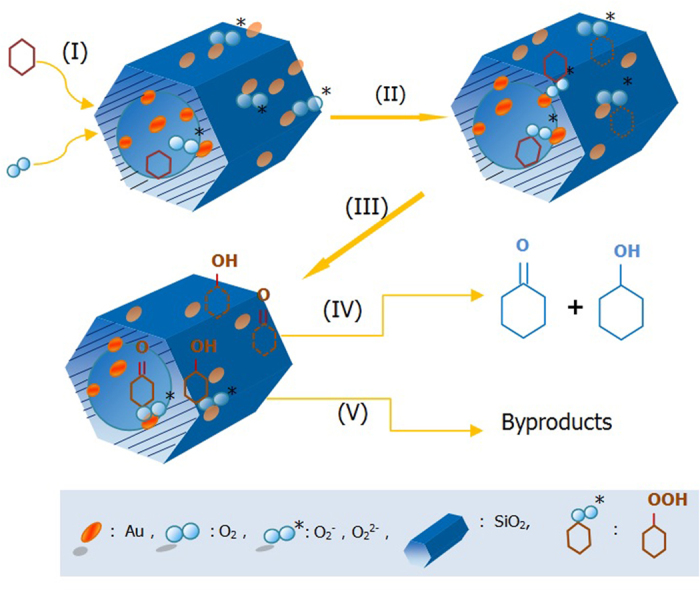
Schematic representation of the mechanism for cyclohexane oxidation on Au/MPTMS-SiO_2_-cal. (I) Diffusion and Adsorption, (II) Reaction to form intermediates, (III) Decomposition of intermediates, (IV) Desorption of K/A oil and (V) Deep oxidation of K/A oil to byproducts.

Since the adsorption and activation of O_2_* molecules is postulated to be a key step in the oxidation reaction^29^, the O_2_ adsorption properties of the Au catalysts were investigated using the O_2_-TPD technique. Figure 6 shows the O_2_-temperature programmed desorption (O_2_-TPD) profiles of pure SBA-15 and catalyst 0.4 *wt*%Au/MPTMS-SiO_2_-cal. It was found that the introduction of Au on mesoporous silica (such as catalyst 0.4 *wt*%Au/MPTMS-SiO_2_-cal) significantly enhanced the oxygen adsorption capacity of silica materials, indicating that Au NPs promote the activation of O_2_ molecules and accelerate the formation of surface active oxygen species, such as O_2_^2−^ and O_2_^−^. The peak below 300 °C is ascribed to desorption of surface-active oxygen species, and the larger desorption peak area at this low temperature range, the higher the catalytic ability for oxidation reaction^30^,^31^. It was observed that on catalyst 0.4 *wt*%Au/MPTMS-SiO_2_-cal the desorption starts at 140 °C and exhibited the highest desorption rate at about 150 °C, while the pure SBA-15 material did not show a distinct desorption peak at this range. At this temperature, the adsorbed surface-active oxygen species such as O_2_^−^ interacted with Au NPs weakly and can react with adsorbed cyclohexane molecules easily to form reactive compound, CyOOH, which is responsible for the chain initiation. These CyOOH further decomposed to form cyclohexanol and cyclohexanone, which diffused from the catalyst surface to be final products K/A oil (as shown in Fig. 7). Part of K/A oil may also be further oxidized to byproducts. The larger amount of surface-active oxygen species released in a certain period of time, the faster the formation of chain initiator, and correspondingly the faster initial reaction rate may be obtained.

**Figure 8:**
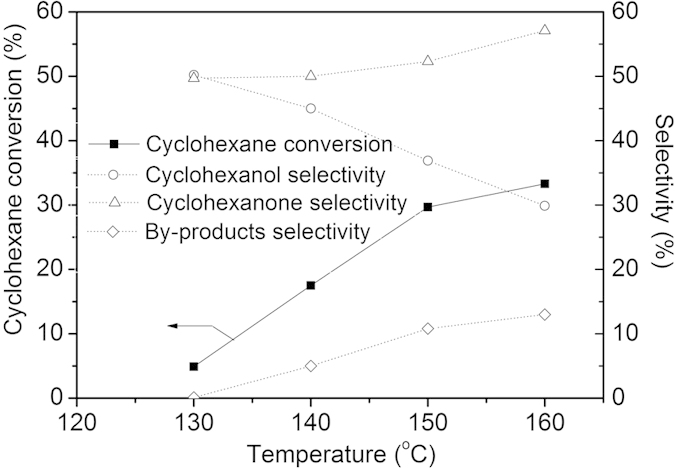
Cyclohexane oxidation over catalyst 0.4 *wt.*%Au/MPTMS-SiO_2_-cal under different reaction temperature. Conditions: 20 ml cyclohexane, 50 mg catalyst, 1 MPa, 2 h.

In order to verify the rationality of above oxygen activation mechanism, the catalytic behavior of catalyst 0.4 *wt.*%Au/MPTMS-SiO_2_-cal under different reaction temperature and reaction kinetics were further studied. The reactions over catalyst 0.4 *wt.*%Au/MPTMS-SiO_2_-cal under different temperatures were conducted and the reaction results are shown in Fig. 8. Based on the O_2_-TPD results, the temperature examined ranged from 130 °C to 160 °C. It was found that the cyclohexane conversion increased with the increase of reaction temperature. At 140 °C, a cyclohexane conversion of 17.5% with a K/A oil selectivity of 95.0% was obtained after two hour of reaction. A further increase of reaction temperature led to a higher cyclohexane conversion (29.7%) at 150 °C with a K/A oil selectivity of 90.1%, which is possibly attributed to the larger amount of weakly adsorbed surface active oxygen species at higher temperature, as confirmed by the O_2_-TPD results. Further increasing the temperature to 160 °C, the cyclohexane conversion was increased to 33.3% but with a dramatically decrease of K/A oil selectivity to 77.0%, suggesting the high temperature accelerates the further oxidation of K/A oil.

**Figure 9:**
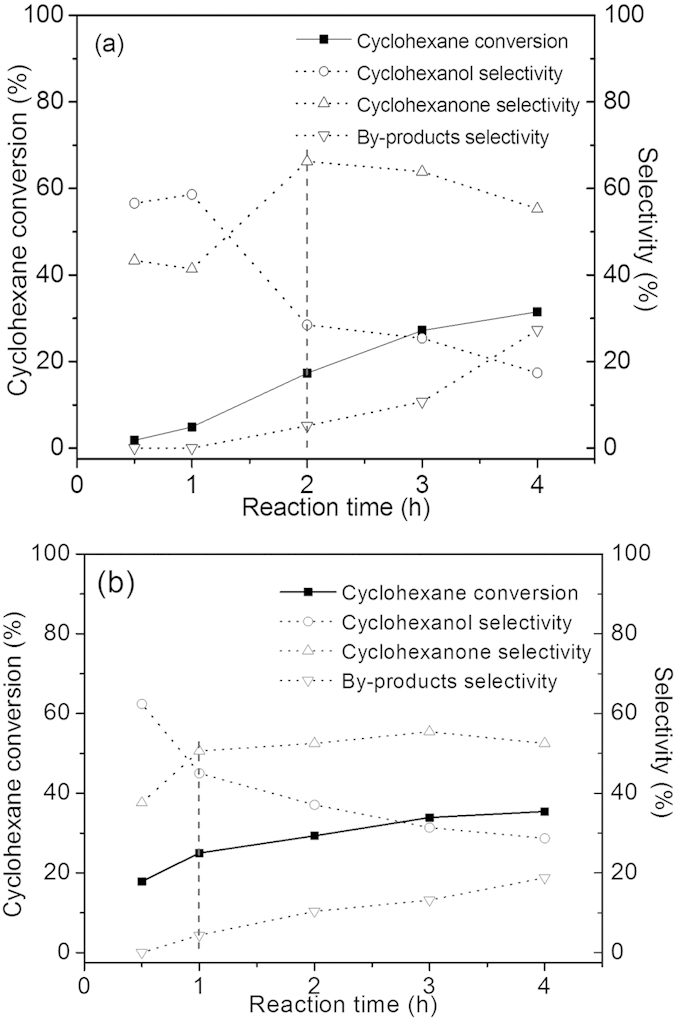
Cyclohexane oxidation over catalyst 0.4 *wt.*%Au/MPTMS-SiO_2_-cal with different reaction time at 140 °C (**a**) and 150 °C (**b**). Conditions: 20 ml cyclohexane, 50 mg catalyst, 1 MPa.

The kinetic studies on catalyst 0.4 *wt.*%Au/MPTMS-SiO_2_-cal at both 140 °C and 150 °C were conducted and the results are shown in Fig. 9. When we compare the cyclohexane conversion in Fig. 9a,b, it can be seen that the initial cyclohexane conversion rate was very slow at 140 °C, less than 5.0% of cyclohexane conversion was obtained after 1 h of reaction. However, the reaction results in Fig. 9b shows that the initial reaction rate was very fast at 150 °C, since the first half an hour of reaction already afforded a cyclohexane conversion of 18.0% with a K/A oil selectivity of >95.0%. It was also observed that the distribution of products exhibited a significant change with the reaction time. The deep oxidation of K/A oil to by-products was observed during the reaction at both140 °C and 150 °C.

The kinetic study results on catalyst 0.4 *wt.*%Au/MPTMS-SiO_2_-cal at 140 °C and 150 °C strongly suggest that further oxidation of cyclohexanol to cyclohexanone and deep oxidation of K/A oil to by-products are the dominating reactions after first one or two hours of reaction. In this process, the formation of by-products is inevitable with the increase of reaction temperature and/or reaction time^14^. Thus, enhancement of initial reaction rate to obtain high cyclohexane conversion in a shorter time is a good choice for improving the catalysis system^32^. Cyclohexane oxidation conducted at 150 °C on catalyst 0.4 *wt.*%Au/MPTMS-SiO_2_-cal (Fig. 9b) exactly provides a very fast initial reaction rate. This high initial reaction rate is due to the highest desorption rate of surface-active oxygen species at about 150 °C, leading to the formation of large amount of reactive compound (CyOOH) for the chain initiation. The fast reaction kinetics and high selectivity for the initial reaction stage would make it possible to built a small reactor and save separation cost, which is of economic significance. The recycling test on catalyst 0.4 *wt.*%Au/MPTMS-SiO_2_-cal for 1 h reaction in 6 cycles have been carried out, and the reaction results are shown in Table S1. The results showed that a slight decrease in conversion occurred after the 2nd run and no obvious activity loss was observed in the following 4 cycles.

## Conclusions

A series of Au catalysts with Au NPs immobilized in the mesoporous silica were prepared via a one-pot synthesis route. The formation mechanism was studied by using different characterization methods. It was found that the gold thiolate complexes [-Au^I^SR-]_n_ were formed in the as-prepared catalyst, which were further reduced to thiolate-passivated gold clusters upon hydrogen or sodium borohydride reduction and were completely decomposed to gold NPs upon calcination. The high-temperature calcined catalyst Au/MPTMS-SiO_2_-cal exhibited a high catalytic activity and remarkably high TOF number (69091 h^−1^) for cyclohexane oxidation because of the presence of larger amount of surface Au (0) sites with low coordination after complete removal of the functional groups. Partial retention of the functional groups in the catalysts deteriorated the catalytic activity through the coordination of active sites with thiol groups. Au NPs were found to play a critical role in the catalytic oxidation of cyclohexane by enhancing the activation of O_2_ molecules and promoting the formation of surface-active oxygen species. The fast reaction kinetics and high selectivity in the initial reaction stage for cyclohexane partial oxidation would be of great industrial significance.

## Methods

### General materials

HAuCl_4_·xH_2_O (Guohua Chemicals), hydrochloric acid (37%, Sinopharm Chemical), triblock co-polymer PEO_20_PPO_70_PEO_20_ (P123, Aldrich), tetraethyl orthosilicate (TEOS, 98%, Acros Organics), mercaptopropyltrimethoxysilane (MPTMS, 97%, Aldrich), absolute ethanol (99.98%, Sinopharm Chemical) and cyclohexane (99.99%, Sinopharm Chemical) were used as received without further purification.

### Preparation of Au NPs catalysts

The Au catalysts were prepared using the method as described previously^14^ with an optimized MPTMS/TEOS ratio of 1/9. The as-synthesized sample after washing and drying was denoted as Au/MPTM*S*-SiO_2_-as. In this work, tri-block copolymer template P123 was removed by either high-temperature calcination or ethanol extraction. The sample obtained after ethanol extraction for three times is denoted as Au/MPTMS-SiO_2_-3et. The reduction of Au species was achieved by high temperature calcination, or H_2_ /NaBH_4_ reduction after the template removal by ethanol.

Calcination was carried out in air at 500 °C for 6 h with a heating rate of 2 °C/min. The reduction of Au species was achieved by auto-reduction and the obtained catalyst is denoted as Au/MPTMS-SiO_2_-cal. The H_2_ reduction was conducted in a H_2_ flow at 250 °C for 2 h with a heating rate of 5 °C/min and the obtained catalyst is designated as Au/MPTMS-SiO_2_-H_2_. The NaBH_4_ reduction was carried out in an ultrapure water solution. Sample Au/MPTMS-SiO_2_-3et was dispersed in ultrapure water, followed by introducing of excessive amount of NaBH_4_ solution under vigorous stirring at room temperature. The white suspension immediately turned to light brown. Finally, the solids were filtered off and dried at 80 °C overnight in a vacuum oven. The obtained catalyst is named Au/MPTMS-SiO_2_-BH_4_.

A range of supported Au NPs catalysts with different Au loadings were also prepared via the one-pot route and reduced by high temperature calcination, designated as *x*Au/MPTMS-SiO_2_-cal *(x* was varied from 0.2 *wt.*% to 1.6 *wt.*%).

### Catalysts characterization

X-ray powder diffraction (XRD) analysis was performed on a XRD-6000 (Shimadzu, Japan) system. Cu *K*α radiation was used with a power setting of 50 kV and 250 mA. The BET surface area of the catalysts was determined in a Micromeritics ASAP2020 and the samples were pretreated at 200 °C for 4 h under vacuum. X-ray photoelectron spectroscopy (XPS) spectra were recorded on an AXIS HIS 165 spectrometer (Kratos Analytical) with a monochromatized Al *Ka* X-ray source. C 1s electron bond energy corresponding to graphitic carbon at 284.5 eV was applied as a calibration binding energy (BE) reference. Elemental analysis of Au in the catalysts was conducted on an Agilent 7500 series inductive-coupled plasma-mass spectrometer (ICP-MS). Before analysis, the solid sample was dissolved by a 2:1 mixture of HNO_3_/HF aqueous solution and filtered. The solid ultraviolet-visible (UV-Vis) spectra analysis was performed on a UV-vis-NIR scanning spectrophotometer (Shimadzu, UV-3101 PC) by using BaSO_4_ as an internal reference. Solid-state magic-angle spinning (MAS) nuclear magnetic resonance (NMR) spectra were obtained on a Bruker DRX400 FT-NMR spectrometer. The single-pulse method was used for ^29^Si spectrum collections while the cross-polarization (CP) technique was used for ^13^C measurements. Transmission electron microscope (TEM) was performed on a JEOL JEM 2010 at 200 kV. O_2_-TPD test was carried out in ChemBET Pulsar TPR/TPD automated chemisorption analyzer (Quantachrome instrument). Prior to each TPD run, the catalyst was calcined to 500 °C in the He flow of 90 mL/min in a quartz reactor. After the temperature was lowered to room temperature, the catalyst was exposed to O_2_ for 30 min under an O_2_ flow of 90 mL/min to allow the adsorption of O_2_ to occur, and then the He was fed into the reactor for 1 h to purge any residual oxygen. After that, the catalyst was heated to 500 °C at a ramping rate of 20 °C/min under He flow. The desorbed oxygen was monitored by the TCD detector.

The metal dispersion on support was calculated by D_*M*_ = (6*n*_*s*_*M*)/(*ρNd*_*p*_), where *n*_*s*_ is the number of gold atoms at the surface per unit area (1.15 × 10^19^ m^−2^ for Au), *M* is the molecular weight of gold (196.97 g mol^−1^), *ρ* is the density of gold (19.5 g cm^−3^), *N* is Avogadro’s number (6.023 × 10^23^ mol^−1^) and *d*_*p*_ is the average gold particle size (determined by HRTEM, admitting that particles are spherical).

### Catalytic testing

All experiments to test the catalytic activity were carried out in a Teflon lined Parr batch reactor. In a general reaction procedure, 20 ml of cyclohexane and 50 mg of solid catalyst were introduced into the reactor. The reactor was sealed, purged with O_2_ and heated to 150 °C. The mixture was allowed to react under an O_2_ pressure of 0.8–1 MPa with a stirring rate of 300 rpm. After 1–2 h of reaction, the reactor was cooled down to room temperature and the products were diluted with ethanol. An excessive amount of triphenylphosphine (Ph_3_P) was added to the reaction mixture to completely reduce the Cyclohexyl hydroperoxide (CHHP), which is an intermediate in the cyclohexane oxidation. The reaction products were analyzed on a gas chromatogram (HP 6890 series GC) with a mass spectrometer detector (HP 5873 mass selective detector) and a capillary column (HP 5MS).

## Additional Information

**How to cite this article**: Wu, P. *et al.* Gold nanoparticles supported on mesoporous silica: origin of high activity and role of Au NPs in selective oxidation of cyclohexane. *Sci. Rep.* **6**, 18817; doi: 10.1038/srep18817 (2016).

## Supplementary Material

Supplementary Information

## Figures and Tables

**Table 1:** Textural properties, Au particle size and metal dispersion of Au catalysts with different Au loading.

Catalysts^a^	*S*_*BET*_(m^2^g^−1^)	*V*_*total*_(cm^3^g^−1^)	Pore diameter (nm)	Au size average^b^ (nm)	Metal dispersion (%)
SBA-15	782	1.0	6.0–10.0	—	—
0.2 *wt.*%Au/MPTMS-SiO_2_-cal	814	0.71	3.5–6.0	2.5	41.5
0.4 *wt.*%Au/MPTMS-SiO_2_-cal	810	0.71	3.5–6.0	2.5	41.6
0.95 *wt.*%Au/MPTMS-SiO_2_-cal	862	0.73	3.5–6.0	2.5	41.5
1.2 *wt.*%Au/MPTMS-SiO_2_-cal	817	0.71	3.5–6.0	3.5	34.0
1.6 *wt.*% Au/MPTMS-SiO_2_-cal	840	0.70	3.5–6.0	9.0	14.6

S_BET_, surface area calculated by the BET method. *V*_*total*_, total pore volume calculated at *P/P*_*o*_ = 0.998. Pore diameter calculated from the adsorption branch using BJH method.

^a^The contents of Au in different catalysts were measured by ICP-MS.

^b^The Au particle size was measured from TEM images.

**Table 2:** Textural properties of the Au catalysts prepared by different reduction methods.

Catalysts	*S*_*BET*_(m^2^g^−1^)	*V*_*total*_(cm^3^g^−1^)	Pore diameter (nm)	Au loading^a^ (*wt.*%)
Au/MPTMS-SiO_2_-3et	642	0.68	6.0–9.5	0.82
Au/MPTMS-SiO_2_-BH_4_	328	0.31	2.5–5.5	0.67
Au/MPTMS-SiO_2_-H_2_	435	0.48	6.0–10.0	0.81
Au/MPTMS-SiO_2_-cal	862	0.73	3.5–6.0	0.95

S_BET_, surface area calculated by the BET method.

*V*_*total*_, total pore volume calculated at *P/P*_*o*_ = 0.998.

Pore diameter calculated from the adsorption branch using BJH method.

^a^Au contents were measured by using ICP-MS.

**Table 3:** Reaction results on different catalysts^a^.

Catalysts^b^	Cyclohexane conversion (mol%)	Products selectivity (mol%)			TOF(1/h)^d^
		Cyclohexanol	cyclohexanone	By-products^c^	
0.2 *wt.*%Au/MPTMS-SiO_2_-cal	16.7	33.4	58.7	7.9	69091
0.4 *wt.*% Au/MPTMS-SiO_2_-cal	29.7	31.5	58.6	9.9	59951
0.95 *wt.*%Au/MPTMS-SiO_2_-cal	27.4	32.9	57.3	9.8	23368
1.2 *wt.*% Au/MPTMS-SiO_2_-cal	28.3	33.8	57.5	8.7	23605
1.6 *wt.*% Au/MPTMS-SiO_2_-cal	16.4	39.2	55.3	5.5	24739
Au/MPTMS-SiO_2_-BH_4_	2.7	62.2	37.7	0.1	2592
Au/MPTMS-SiO_2_-H_2_	15.8	53.0	43.9	3.1	12181

^a^Reaction conditions: 20 ml cyclohexane, 50 mg catalyst, 150 ^o^C, 1 MPa, 2 h;

^b^Au loading was measured by ICP-MS;

^c^By-products are mainly ring-opened acids such as n-butyric, succinic, glutaric and adipic acid;

^d^Moles of K/A oil produced on per mole of surface Au per hour.
