# Supplementary material for: Gold nanoparticles supported on mesoporous silica: origin of high activity and role of Au NPs in selective oxidation of cyclohexane
Source: Sci Rep. 2016 Jan 5;6:18817. doi: 10.1038/srep18817 (PMC4700469; doi:10.1038/srep18817)
Supplement: Supplementary Information [file srep18817-s1.doc]

**Supplementary Information**

**Gold nanoparticles supported on mesoporous silica: origin of high activity and role of Au NPs in selective oxidation of cyclohexane**

Pingping Wu^1^,*, Peng Bai^1^ , Zifeng Yan^1^, George X. S. Zhao ^2^,*

^1^State Key Laboratory of Heavy Oil Processing, PetroChina Key Laboratory of Catalysis, School of Chemical Engineering, China University of Petroleum, Qingdao, China, 266580，^2^ School of Chemical Engineering, The University of Queensland, St Lucia, 4072.

E-mail :wupp@upc.edu.cn (P. P. Wu), [george.zhao@uq.edu.au](mailto:george.zhao@uq.edu.au) (George X. S. Zhao )

The N_2_ adsorption/desorption isotherms of different Au loading catalysts are shown in Figure S1. It is seen from FigureS1 that all catalysts displayed a type IV isotherm with a hysteresis loop. The pore size distribution centered in the range of 4 - 6 nm; however, the pore size distribution became wider with the increase of Au loading. This is due to the distortion effect caused by the immobilization of Au NPs in the pore surface.

**Figure S1** N_2_ adsorption/desorption isotherms and the corresponding pore size distribution of catalysts (a) 0.2 *wt.*%Au/MPTMS-SiO_2_-cal, (b) 0.4 *wt.*%Au/MPTMS-SiO_2_-cal, (c) 0.95 *wt.*%Au/MPTMS-SiO_2_-cal and (d) 1.2 *wt.*%Au/MPTMS-SiO_2_-cal and (e) 1.6 *wt.*% Au/MPTMS-SiO_2_-cal.

The evidence for the incorporation of organosiloxanes into the silica framework of catalysts Au/MPTMS-SiO_2_-BH_4_ and Au/MPTMS-SiO_2_-H_2_ was obtained from ^29^Si and ^13^C CP MAS NMR results shown in Figure S2. In ^29^Si MAS NMR spectra, the resonances at about -65 ppm and -56 ppm which are attributed to the resonances of T_3_ and T_2_ (*T*_x_ =(SiO)_x_(OH)_3-x_SiC) for organosiloxane [^1^](#_ENREF_1), were observed on sample Au/MPTMS-SiO_2_-*as,* catalysts Au/MPTMS-SiO_2_-BH_4_ and Au/MPTMS-SiO_2_-H_2_. The dominant T_3_ resonance over T_2_ resonance confirmed the incorporation of the mercaptopropyl groups in the as-synthesized sample Au/MPTMS-SiO_2_-*as*, catalysts Au/MPTMS-SiO_2_-BH_4_ and Au/MPTMS-SiO_2_-H_2_. However, on catalyst Au/MPTMS-SiO_2_-cal, no T_3_ or T_2_ resonance peaks for organosiloxane were observed because of the complete removal of functional groups during high temperature treatment. From ^13^C CP MAS NMR spectra, three resonances at 11 ppm, 22 ppm, and 26 ppm assigned to C^1^, C^2^ and C^3^ of mercaptopropyl group (-C^1^H_2_-C^2^H_2_-C^3^H_2_-SH) were observed on catalysts Au/MPTMS-SiO_2_-BH_4_ and Au/MPTMS-SiO_2_-H_2_, confirming the presence of organosiloxanes in these catalysts. The intensities of peaks due to C^1^, C^2^ and C^3^ of mercaptopropyl group on catalyst Au/MPTMS-SiO_2_-H_2_ were lower than those of as-synthesized and NaBH_4_ reduced catalyst, due to the partial decomposition of functional groups during H_2_ reduction (250 ^o^C).

Figure S2. ^29^Si (left) and ^13^C (right) MAS NMR spectra of catalysts (a) Au/MPTMS-SiO_2_-as, (b) Au/MPTMS-SiO_2_-BH_4_, (c) Au/MPTMS-SiO_2_-H_2_ and (d) Au/MPTMS-SiO_2_-cal.

Recycling test with repeated use of catalyst 0.4 wt.% Au/MPTMS-SiO_2_-cal in six reaction cycles were carried out. The catalyst was filtrated from the reaction system after 1 h reaction and washed thoroughly with ethanol, followed by drying at 80 ^o^C overnight and then subjected to the next cycle. The recycling results are shown in Table S1. A slight decrease in conversion occurred after the 2nd run on catalyst 0.4 wt.% Au/MPTMS-SiO_2_-cal and no obvious activity loss was observed in the following 4 cycles.

Table S1 Recycling test on catalyst 0.4 wt.% Au/MPTMS-SiO_2_-cal^a^

| 0.4 *wt.*% Au/MPTMS-SiO_2_-cal | Cyclohexane conversion  (mol%) | Products selectivity (mol%) | | |
| --- | --- | --- | --- | --- |
|  |  | Cyclohexanol | cyclohexanone | By-products *^b^* |
| (1^st^ use) | 25.2 | 45.0 | 50.6 | 4.4 |
| (2^nd^ use) | 23.6 | 46.6 | 49.4 | 4.0 |
| (3^rd^ use) | 22.6 | 46.4 | 48.6 | 5.0 |
| (4^th^ use) | 23.0 | 46.4 | 48.8 | 4.8 |
| (6^th^ use) | 23.5 | 46.2 | 48.6 | 5.2 |

*^a^* Reaction conditions: 20ml cyclohexane, 50mg catalyst, 150 ^o^C, 1MPa, 1h;

*^b^* By-products are mainly ring-opened acids such as n-butyric, succinic, glutaric and adipic acid;

**Reference**

(1) Liu, J.; Yang, Q. H.; Zhang, L.; Jiang, D. M.; Shi, X.; Yang, J.; Zhong, H.; Li, C. *Adv. Funct. Mater.* **2007**, *17*, 569.

(2) Lim, M. H.; Blanford, C. F.; Stein, A. *Chem. Mat.* **1998**, *10*, 467.

(3) Feng, X.; Fryxell, G. E.; Wang, L. Q.; Kim, A. Y.; Liu, J.; Kemner, K. M. *Science* **1997**, *276*, 923.
